# Magnesium-related gene ITGAL: a key immunotherapy predictor and prognostic biomarker in pan-cancer

**DOI:** 10.3389/fphar.2024.1464830

**Published:** 2024-11-13

**Authors:** Fengjie Lin, Hanxuan Yang, Zongwei Huang, Ying Li, Qin Ding, Yunbin Ye, Sufang Qiu

**Affiliations:** ^1^ Department of Radiation Oncology, Clinical Oncology School of Fujian Medical University, Fujian Cancer Hospital, Fujian, China; ^2^ Laboratory of Immuno-Oncology, Fujian Cancer Hospital and Fujian Medical University Cancer, Hospital, Fuzhou, China; ^3^ Fujian Key Laboratory of Translational Cancer Medicine, Fuzhou, China

**Keywords:** ITGAL, pan-cacner, immunothearpy, magnesium-related, HNSCC

## Abstract

**Background:**

Integrin subunit alpha L (ITGAL) is crucial for activating CD8^+^ T cells through magnesium-mediated immune synapse formation and specific cytotoxicity. ITGAL might exert an important function in the growth and transformation of cancer.

**Methods:**

Our study comprehensively analyzed ITGAL expression across various cancers, validated by Immunochemistry (IHC) in the laboratory. ITGAL showed prognostic significance in pan-cancer patients, correlated with clinical features, and associated with specific signaling pathways. We also observed a relationship between ITGAL and immune cell infiltration. In HNSCC, ITGAL demonstrated prognostic value and potential implications for immunotherapy response and novel drug targets.

**Results:**

ITGAL expression linked to tumor prognosis across 27 cancers. Elevated ITGAL correlated with good prognosis in CESC, LUAD, SARC, HNSCC, and SKCM. ITGAL involved in immune regulation pathways and showed positive correlation with immune cell infiltration. ITGAL associated with CD8^+^ T cell infiltration. And high ITGAL expression in CD8^+^ T cells and NK cells. In HNSCC, ITGAL linked to favorable prognosis and sensitivity to immunotherapy. Predicted potential drugs for HNSCC.

**Conclusion:**

ITGAL is remarkably associated with CD8+T cells and crucial in the tumor immune microenvironment of pan-cancer. Furthermore, our findings may provide a targeted anti-tumor strategy for ITGAL by influencing the tumor immune microenvironment.

## Introduction

Integrin alpha L chain encoded by integrin subunit alpha L (ITGAL) is critically involved in intercellular adhesion between leukocytes by binding to intercellular adhesion molecules 1–3 (ICAMs 1–3) (Corbi et al., 1988; Hickman et al., 2022). Moreover, previous studies have demonstrated that the LFA-1 (Lymphocyte Function-associated Antigen 1) encoded by ITGAL is crucial in the inflammatory response (Whitcup et al., 1999), which includes cytotoxic T cell dependent killing, antibody mediated killing by granulocytes, monocytes and leukocyte-endothelial cell interaction. It also promotes the cytotoxicity of natural killer (NK) cells (Barber et al., 2004).

LFA-1 encoded by ITGAL has recently been found to be involved in CD8^+^ T cell activation. To acquire active conformation, magnesium is required by LFA-1 on CD8+T cells. As a result calcium flux, metabolic reprogramming, signal transduction, immune synapse formation, and specific cytotoxicity are enhanced (Lotscher et al., 2022).

ITGAL belongs to the integrin family, and the integrin family plays a critical role in the control of angiogenesis and lymphangiogenesis, fundamental processes crucial for the advancement and spread of tumors. The exploration of integrins as therapeutic targets shows significant potential in the realm of cancer treatment (Avraamides et al., 2008). In T cell-mediated immunity, integrins are pivotal in governing lymphocyte recirculation, activating T cells, and delineating distinct subsets of T cells and antigen-presenting cells (Pribila et al., 2004). Integrins are also pivotal in the processes of tumour stemness, metastasis, and drug tolerance. A comprehensive understanding of their regulatory mechanisms holds the promise of unveiling innovative therapeutic strategies aimed at enhancing tumor responsiveness to treatments while mitigating metastatic characteristics (Seguin et al., 2015). Existing studies have shown that ITGAL can affect the prognosis and survival of tumors such as melanoma and gastric cancer through tumor immunity (Zhang et al., 2022; Deng et al., 2023). Furthermore, ITGAL is associated with poor prognosis in ovarian cancer (Wu A. et al., 2020), while it also suggests better prognosis and inhibits tumor proliferation in NSCLC (Wang et al., 2023). The above studies reflect the heterogeneity of the impact of ITGAL on different cancers, and there are no studies on pan-cancer analysis of ITGAL, which triggered our interest in doing pan-cancer analysis of ITGAL.

The objective of this research is to evaluate the correlation between ITGAL expression and prognosis in various cancer types, as well as the impact on the immune microenvironment. At the same time, we hope to propose a targeted anti-tumor strategy for ITGAL by regulating the tumor immune microenvironment (TME) and find corresponding anti-cancer drugs.

## Methodology

### Immunochemistry (IHC)

A semiquantitative integration method was utilized to evaluate the intensity of IHC staining in eight different types of cancer tissues and adjacent normal tissues. Images of the stained tissues were captured using a microscope (3DHISTECH, Hungary) at ×20 magnification. Protein expression levels were quantified using the histochemistry score (H-score), calculated with the following formula: H-score = (proportion of cells exhibiting low intensity × 1) + (proportion of cells exhibiting middle intensity × 2) + (proportion of cells exhibiting high intensity × 3).

### Database

The TCGA database was utilized to retrieve the RNA-seq data of tumor and paired-healthy tissues (https://portal.gdc.cancer.gov/). Data from UCSC’s XENA database were acquired from the TCGA and GTEx for unpaired analyses (https://xenabrowser.net/datapages/). TCGA-HNSC collection (https://portal.gdc.cancer.gov/projects/TCGA-HNSC) was accessed for gathering clinical information on head and neck squamous cell carcinoma (HNSCC).

### Exploring the relationship between ITGAL and clinical features

To analyze the data, a univariate COX regression model was built using the “survival” package in R. The predictive significance of ITAGL was assessed using four clinical endpoints: OS (overall survival), DSS (disease-specific survival), DFS (disease-free interval), and PFS (progression-free interval). In the evaluation of prognostic markers, we conducted an analysis that involved calculating hazard ratios (HR), 95% confidence intervals, and p-values. To determine the statistical significance in this study, we utilized a significance level of p < 0.05. This threshold helped us identify associations that were unlikely to occur by chance and confirmed the statistical significance of our findings. To examine the association between the clinical stage and the expression of ITGAL, we performed correlation analysis utilizing the R packages “limma” and “ggpubr.”

### Immune infiltration analysis

We used the “limma” package in R to assess the expression levels of these genes as well as examining their correlation coefficients using the Pearson statistical method. Afterwards, we employed the “ESTIMATE” package to compute the StromalScore, ImmuneScore, and ESTIMATE scores for a dataset consisting of 10,180 tumor samples across 44 different tumor types. To analyze the statistical correlation between gene expression and immune infiltration scores in each tumor, we utilized the “psych” package in R. This analysis revealed significant associations between gene expression and immune infiltration scores. To further validate these findings, immune cell infiltration data for 33 different types of cancer were retrieved from the TIMER 2.0 database (http://timer.cistrome.org) for comparison (Li et al., 2020). The visualization of the results was accomplished using the R packages “reshape2” and “RColorBrewer.”

### Biomarker Exploration of Solid Tumors

The survival prognosis of associated genes could be assessed through Biomarker Exploration of Solid Tumors (BEST) (http://www.rookieutopia.com) by mapping the survival curve utilizing pan-cancer samples, including GBM (CGGA325, CGGA693), LGG (CGGA301, CGGA325, CGGA693, and TCGA), CESC (TCGA), LUAD [GSE72094 (Schabath et al., 2016), GSE41271 (Sato et al., 2013; Riquelme et al., 2014; Girard et al., 2016; Parra et al., 2016), and GSE26939 (Wilkerson et al., 2012)], HNSCC [GSE65858 (Wichmann et al., 2015)], SKCM (GSE53118 (Mann et al., 2013; Barter et al., 2014), GSE54467 (Jayawardana et al., 2015), GSE1900113 (Farshidfar et al., 2022), and TCGA), SARC [GSE21257 (Buddingh et al., 2011) and TCGA]. The ITGAL’s prognostic value in pan-cancer in terms of overall survival (OS) and post-progression survival (PPS) was assessed utilizing this database. The log-rank p-value and the hazard ratio (HR) with 95% confidence intervals were also estimated. *p* < 0.05 was taken as statistically significant.

### Enrichment analysis and analysis of genomic heterogeneity as well as stemness

The biological roles of ITGAL in tumors were determined through Gene set enrichment analysis (GSEA). We downloaded the gene ontology (GO) from the official GSEA website (https://www.gsea-msigdb.org/gsea/downloads.jsp). R-packages “clusterProfiler”were employed for functional analysis.

### Single-cell sequencing analysis

A single-cell RNA-sequencing (scRNA-seq) data of BLCA [GSE145281_aPDL1 (Yuen et al., 2020)], CRC [GSE136394 (Lu et al., 2019), GSE139555 (Wu T. D. et al., 2020; Banta et al., 2022), and GSE146771 10X (Zhang et al., 2020)], HNSCC [GSE103322 (Puram et al., 2017), and GSE139324 (Cillo et al., 2020; Ruffin et al., 2021)], and SKCM [GSE72056 (Tirosh et al., 2016), GSE115978_aPD1 (Jerby-Arnon et al., 2018), and GSE120575_aPD1aCTLA4 (Sade-Feldman et al., 2018)] were studied based on the Tumor Immune Single Cell Hub (TISCH) database (Sun et al., 2021). The immune cells were annotated into five clusters: NK cells, B cells, CD8+T cells, monocyte or macrophage (Mono/Macro), and conventional CD4 T cells (CD4Tconv).

### Drug targeted therapy and Candidate drug prediction and analysis of genomic heterogeneity as well as stemness

A gene-specific targeted therapy analysis was conducted through the BEST website, focusing on the impact of the key gene (ITGAL) on immune modulation therapy in the Cho (2020) and Hwang (2020) cohorts. Additionally, a drug prediction analysis was carried out to explore potential targeted therapy options for Head and Neck Squamous Cell Carcinoma (HNSCC), utilizing datasets such as GSE117973, E_MTAB_8588, TCGAHNSC, GSE75538, and GSE65858. We obtained and analyzed the MSI scores, TMB scores, and RNA-seq data for a specific tumor from the sangerbox3.0 platform. (Shen et al., 2022).

## Result

### Expression of ITGAL in pan-cancer

**FIGURE 1:**
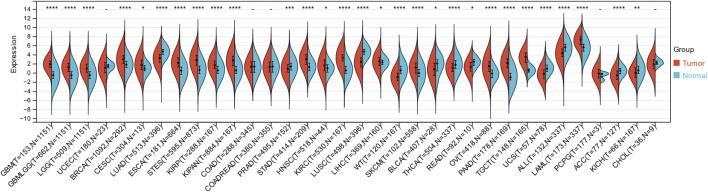
The expression of ITGAL in pan-cancer and healthy tissues (TCGA + GTEx). (-, *p* ≥ 0.05; *, *p* < 0.05; **, p < 0.01; ***, *p* < 0.001; ****, *p* < 0.0001).

Through the integration and exploration of the TCGA and GTEx databases, we acquired the expression levels of ITGAL across multiple cancer types, providing evidence that ITGAL is overexpressed in 18 different tumors such as GBM, GBMLGG, LGG, BRCA, CESC, ESCA, STES, KIRP, KIPAN, STAD, HNSCC, KIRC, LIHC, SKCM, OV, PAAD, TGCT, LAML (Figure 1; Table 1). We also observed significant downregulation of ITGAL expression in 11 tumors, such as LUAD, PRAD, LUSC, WT, BLCA, THCA, READ, UCS, ALL, ACC, KICH (Figure 1; Table 1).

**TABLE 1:** Differential expression of ITGAL in cancer and adjacent normal tissues.

Tumor type	Tumor (Mean ± SD)	Normal (Mean ± SD)	p-value	Regulation status
GBM	1.95 ± 1.09	−0.38 ± 1.29	2.40E-61	Overexpression
GBMLGG	1.29 ± 1.30	−0.38 ± 1.29	3.70E-117	Overexpression
LGG	1.10 ± 1.30	−0.38 ± 1.29	8.90E-84	Overexpression
BRCA	2.91 ± 1.36	1.78 ± 1.13	6.20E-40	Overexpression
CESC	1.73 ± 1.54	0.92 ± 1.22	0.05	Overexpression
ESCA	2.20 ± 1.53	0.55 ± 1.27	3.70E-35	Overexpression
STES	2.77 ± 1.54	0.76 ± 1.31	9.20E-109	Overexpression
KIRP	1.81 ± 1.28	0.74 ± 1.38	3.40E-16	Overexpression
KIPAN	2.57 ± 1.61	0.74 ± 1.38	1.80E-37	Overexpression
STAD	3.02 ± 1.48	1.43 ± 1.23	1.10E-33	Overexpression
HNSC	1.60 ± 1.68	1.06 ± 1.14	0.01	Overexpression
KIRC	3.30 ± 1.25	0.74 ± 1.38	7.30E-58	Overexpression
LIHC	2.53 ± 1.24	2.34 ± 0.74	0.01	Overexpression
SKCM	1.22 ± 1.73	−0.02 ± 1.16	4.30E-12	Overexpression
OV	1.37 ± 1.68	−0.11 ± 1.22	1.50E-15	Overexpression
PAAD	2.08 ± 1.53	−0.67 ± 1.40	1.10E-38	Overexpression
TGCT	3.38 ± 1.42	0.62 ± 0.66	2.80E-45	Overexpression
LAML	7.06 ± 1.26	5.57 ± 1.24	2.10E-30	Overexpression
LUAD	3.17 ± 1.22	4.68 ± 0.77	6.00E-75	Downregulation
PRAD	1.00 ± 1.27	1.46 ± 1.22	1.30E-04	Downregulation
LUSC	2.33 ± 1.41	4.68 ± 0.77	1.70E-111	Downregulation
WT	−0.92 ± 1.26	0.74 ± 1.38	2.90E-21	Downregulation
BLCA	0.87 ± 1.84	1.66 ± 1.87	0.01	Downregulation
THCA	1.24 ± 1.39	1.95 ± 1.39	5.60E-12	Downregulation
READ	1.27 ± 1.39	2.36 ± 1.04	0.02	Downregulation
UCS	−0.10 ± 1.53	1.13 ± 0.98	1.80E-07	Downregulation
ALL	4.26 ± 1.53	5.57 ± 1.24	2.30E-17	Downregulation
ACC	−0.51 ± 1.52	0.44 ± 0.84	2.70E-07	Downregulation
KICH	0.06 ± 1.34	0.74 ± 1.38	2.60E-03	Downregulation

### Immunochemistry (IHC)

**FIGURE 2:**
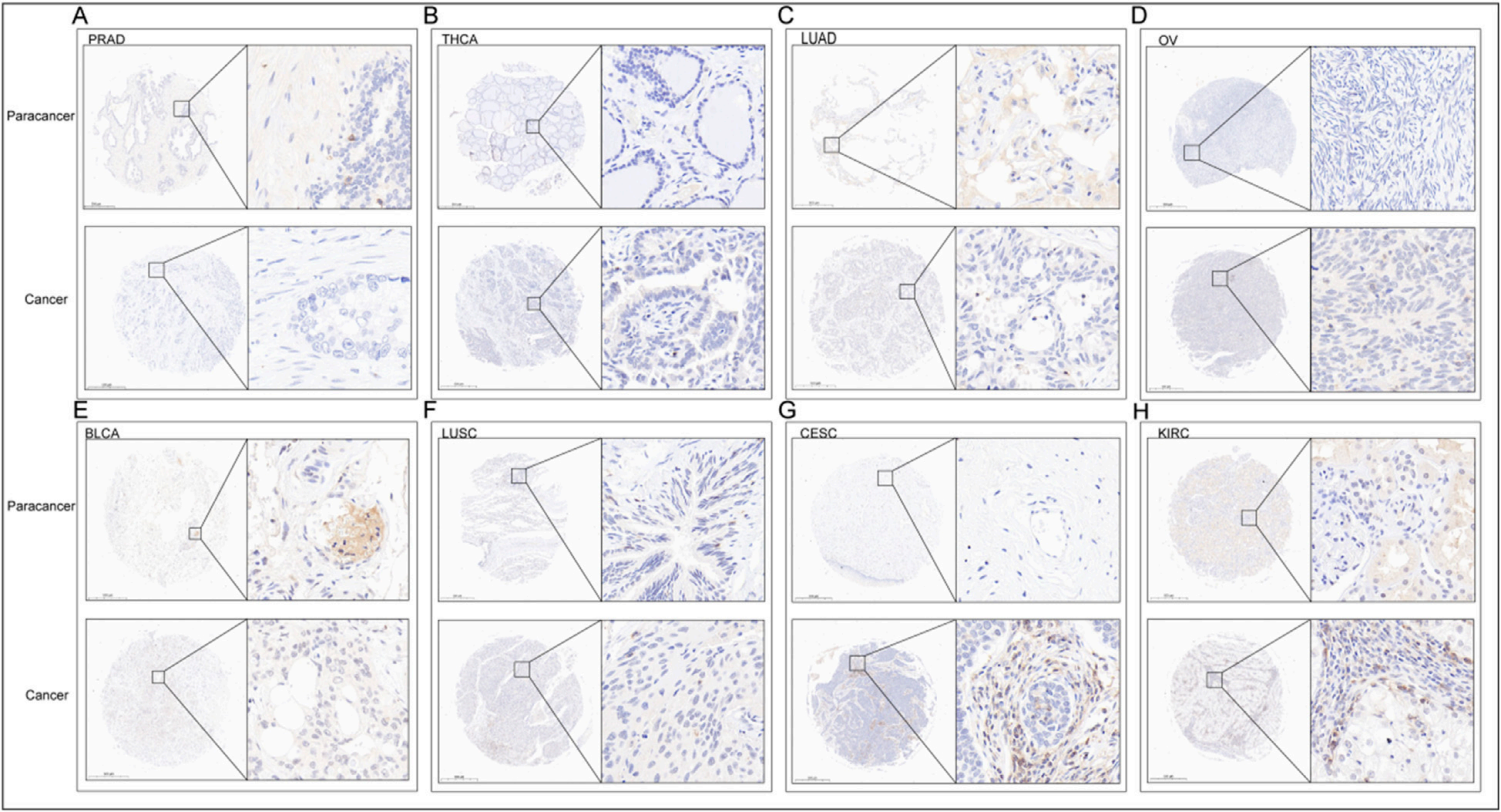
IHC of PRAD **(A)**, THCA **(B)**, LUAD **(C)**, OV **(D)**, BLCA **(E)**, LUSC **(F)**, CESC **(G)**, KIRC **(H)**. The upper row is the paracancerous tissue, and the lower row is the cancer tissue in each unit.

In order to verification the different expression of ITGAL in tumor and peritumoral tissues. We observed variations in the levels of ITGAL expression between seven types of cancer and their corresponding paracancerous tissues (Thyroid carcinoma, Prostate adenocarcinoma, Lung squamous cell carcinoma, Lung adenocarcinoma, Cervical squamous cell carcinoma and endocervical adenocarcinoma, Ovarian serous cystadenocarcinoma, Bladder Urothelial Carcinoma) (Figures 2A–H). The conclusions reached were the same as the previous analysis and showed that the expression of ITGAL in prostate cancer, thyroid cancer, bladder cancer, lung squamous cell carcinoma, and lung adenocarcinoma are highly expressed in adjacent cancer tissues, on the other hand, ovarian cancer, renal clear cell carcinoma, and cervical cancer are highly expressed in cancer tissues Table 2.

**TABLE 2:** Numerical values related to ICH experimental results.

Cancer type	Tissue type	Positive cells, %	Positive cells density, number/mm^2^	Mean density	H-score	IRS
PRAD	paracancerous tissues	0.99%	17	1.9798	2.65	0
	cancerous tissues	3.12%	292	1.0625	6.77	0
THCA	paracancerous tissues	7.22%	117	1.2745	19.42	3
	cancerous tissues	5.00%	183	1.9055	13.71	0
LUAD	paracancerous tissues	21.97%	110	0.5126	39.06	2
	cancerous tissues	6.81%	148	1.0810	16.29	2
OV	paracancerous tissues	0.82%	34	2.5953	2.30	0
	cancerous tissues	7.63%	369	0.6443	12.51	2
BLCA	paracancerous tissues	50.49%	201	0.8031	114.85	6
	cancerous tissues	21.70%	434	0.7575	35.27	2
LUSC	paracancerous tissues	14.47%	252	1.0096	36.73	3
	cancerous tissues	13.51%	362	0.7841	30.46	2
CESC	paracancerous tissues	10.77%	112	2.8235	30.67	3
	cancerous tissues	21.34%	1,017	0.7500	47.63	2
KIRC	paracancerous tissues	29.80%	567	0.4881	54.88	4
	cancerous tissues	43.31%	998	0.6414	83.56	4

### Survival analysis

**FIGURE 3:**
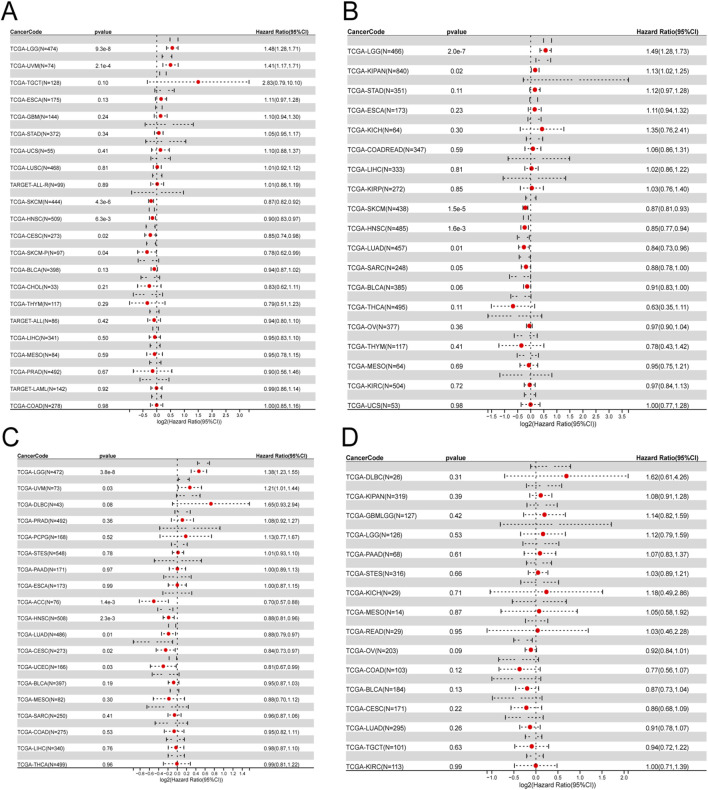
Univariate COX regression analysis was performed to assess the association between ITGAL and O), PFS, DFS, DSS. **(A)** Correlation between ITGAL expression and OS; **(B)**, DSS; **(C)**, PFS; **(D)**, DFS. OS, overall survival; DSS, disease-specific survival; DFS, disease-free survival; PFS, progression-free survival.

By conducting survival analysis in four domains—Overall Survival (OS), Disease-Specific Survival (DSS), Progression-Free Survival (PFS), and Disease-Free Survival (DFS)—we discovered the prognostic significance of ITGAL across various cancer types. Applying Cox regression model analysis, we found a correlation between elevated ITGAL expression and a higher likelihood of decreased overall survival (OS) in patients diagnosed with five specific types of tumors: GBMLGG, LGG, KIPAN, UVM, and LAML (as demonstrated in Figure 3A). Conversely, ITGAL acted as a protective factor in six cancer types, namely CESC, LUAD, LARC, HNSCC, SKCM-P, SKCM, and SKCM-M. Additional investigation revealed a notable correlation between the expression of ITGAL and Disease-Specific Survival (DSS) across various carcinoma categories, such as GBMLGG, LGG, KIPAN, UVM, BRCA, CESC, LUAD, HNSCC, SKCM-P, SKCM, and SKCM-M as depicted in Figure 3B. Furthermore, a Univariate Cox regression model was employed to investigate the correlation between ITGAL expression and Progression-Free Survival (PFS) in various cancer types. In nine types of tumors, namely UVM, UCEC, BRCA, CESC, HNSCC, SKCM, SKCM-M, ACC, and CHOL, the study found a significant correlation between the expression of ITGAL and a positive prognosis (as shown in Figure 3C). Additionally, high expression of ITGAL was indicative of lower Disease-Free Survival (DFS) specifically in the case of BRCA (as depicted in Figure 3D).

### The association between ITGAL and pathological grades

**FIGURE 4:**
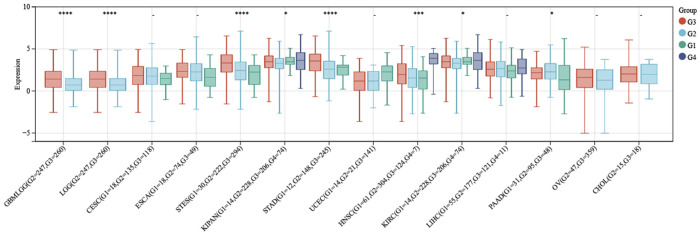
Expression levels of ITGAL at distinct pathological grade in pan-cancer.

After conducting a more comprehensive analysis of ITGAL expression levels across different pathological grades in the context of pan-cancer, we observed a notable discrepancy in seven tumor types in Figure 4. In this study, we employed R software to compute the expression variations of genes within each tumor across samples with different clinical stages. To assess the significance of the differences between the two groups, we conducted a statistical analysis using the unpaired Student's t-test and analysis of variance (ANOVA). Difference test for multiple groups of samples in clinical stage analysis, we observed significant differences in seven types of tumors such as STES (Stage I = 76, II = 201, III = 230, IV = 57) (*p* = 2.4e-3), KIPAN (Stage I = 464, II = 107, III = 189, IV = 103) (*p* = 9.5e-4), STAD (Stage I = 58, II = 121, III = 169, IV = 41) (*p* = 3.3e-3), THYM (Stage I = 36, II = 61, III = 14, IV = 6) (*p* = 2.3e-4), THCA (Stage I = 283, II = 52, III = 112, IV = 55) (*p* = 6.5e-3), SKCM (Stage II = 66, III = 26, IV = 3) (*p* = 0.02), CHOL (Stage I = 19, II = 9, IV = 7) (*p* = 0.02). And we also found differences in eight types of tumors such as GBMLGG (G2 = 247, G3 = 260) (*p* = 6.2e-7), LGG (G2 = 247,G3 = 260) (*p* = 6.2e-7), STES (G1 = 30,G2 = 222,G3 = 294) (*p* = 4.0e-11), KIPAN (G1 = 14, G2 = 228, G3 = 206, G4 = 74) (*p* = 0.02), STAD (G1 = 12,G2 = 148,G3 = 245) (*p* = 1.2e-9), HNSCC(G1 = 61,G2 = 304,G3 = 124,G4 = 7) (*p* = 6.1e-4), KIRC (G1 = 14,G2 = 228,G3 = 206,G4 = 74) (*p* = 0.02), AAD (G1 = 31,G2 = 95,G3 = 48) (*p* = 0.04) in Difference test for multiple groups of samples in stage pathological analysis.

### ITGAL exhibits a strong association with immune infiltration and immune checkpoint

**FIGURE 5:**
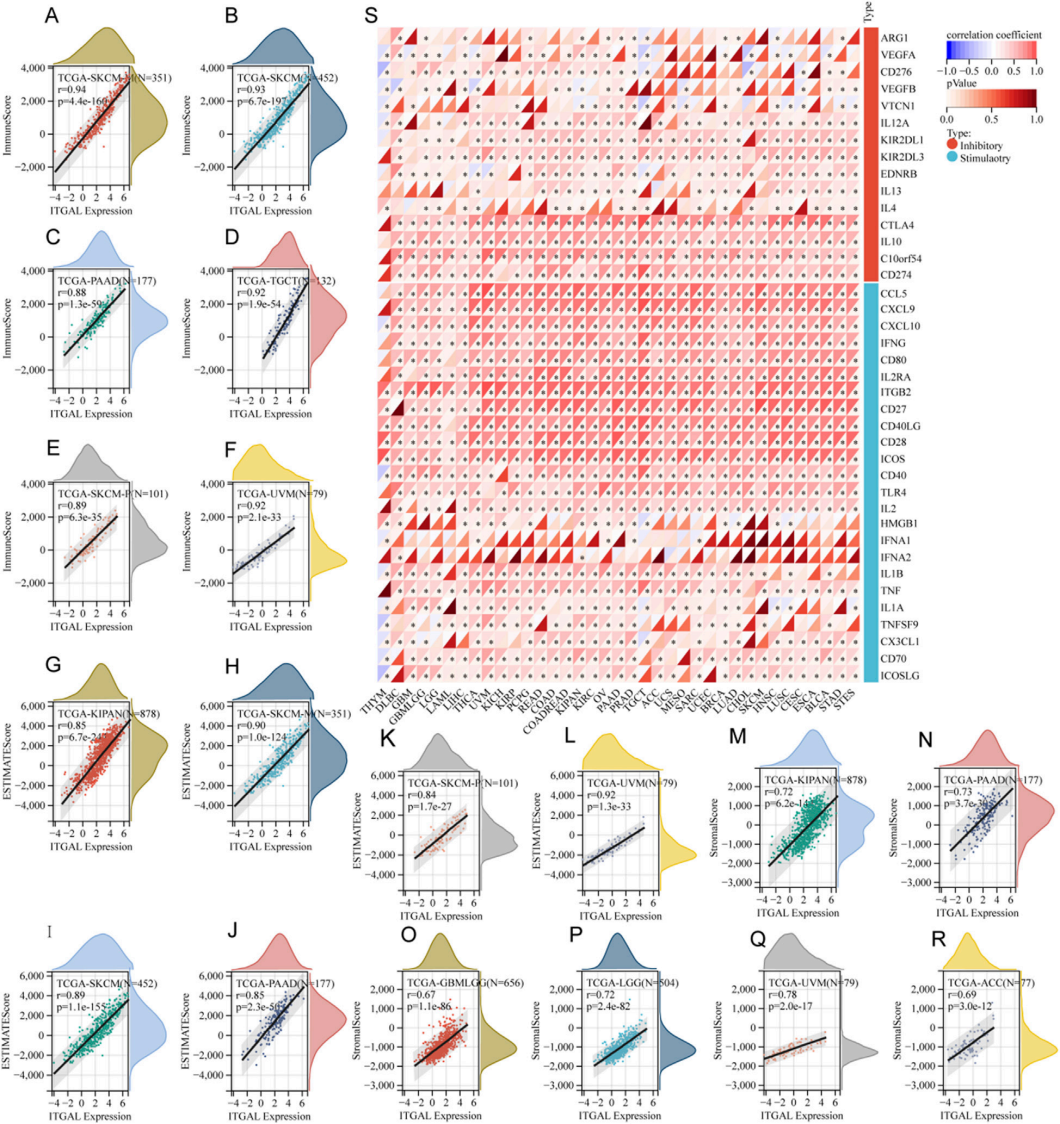
The relationship between ITGAL expression and immune infiltration as well as immune checkpoints. **(A–F)** Correlation of ITGAL expression with ImmuneScore; **(S)** Correlation of ITGAL expression with immune checkpoint-related genes; **(G–L)** Correlation of ITGAL expression with EstimateScore; **(M–R)** Correlation of ITGAL expression with StromalScore.

The study focused on investigating the role of ITGAL in the TME, its relationship with immune infiltration in different cancer types. Specifically, the researchers analyzed the correlation between ITGAL and three distinct immune scores. The results of this analysis are showcased in Figures 5A–R, which highlight the six most notable correlations between ITGAL and the diverse immune scores in specific cancer types. According to the ImmuneScore revealed that the expression of ITGAL in SKCM, SKCM-M, PAAD, TGCT, SKCM-P and UVM was positively correlated with immune infiltration (Figures 5A–F). According to the EstimateScore, the analysis suggests that the expression of ITGAL is correlated with increased levels of immune infiltration in several tumor types, including KIPAN, SKCM-M, SKCM, PAAD, SKCM-P, and UVM (Figures 5G–L). This trend was also suggested by the StromalScore, ITGAL expression in GBMLGG, LGG, KIPAN, PAAD, UVM and ACC was significantly positive correlated with immune infiltration (Figures 5M–R). Despite numerical variations in the three scores, there was a consistent overall trend indicating that ITGAL plays a significant regulatory role in the tumor microenvironment to some extent in malignant conditions. In our study, we undertook a thorough analysis to investigate the potential correlation between ITGAL expression and 60 genes related the immune checkpoint pathway in diverse cancer types. The findings, depicted in Figure 5S, revealed significant correlations between ITGAL and a wide range of immunosuppressive/immunostimulatory genes present in pan-cancer. Specifically, ITGAL exhibited strong positive correlations with most immune checkpoint pathway genes. These results indicate that ITGAL is closely associated with immune checkpoint genes and predominantly facilitates the infiltration of immune cells.

### ITGAL-related GSEA

The GSEA algorithm analysis was carried out in pan-cancer to elucidate the underlying physiological processes that might be mediated by ITGAL and subsequently, six tumors with similar results were selected (Supplementary Figure S1). It was found that ITGAL participated in pan-cancer immune regulation-related pathways, particularly in leukocyte-mediated cytotoxicity, lymphocyte-mediated immunity, adaptive immune response, cytokine signaling in the immune system, and antigen processing and presentation. It was highlighted by these findings that ITGAL is crucially involved in tumor immunity.

### Immune infiltration analysis

**FIGURE 6:**
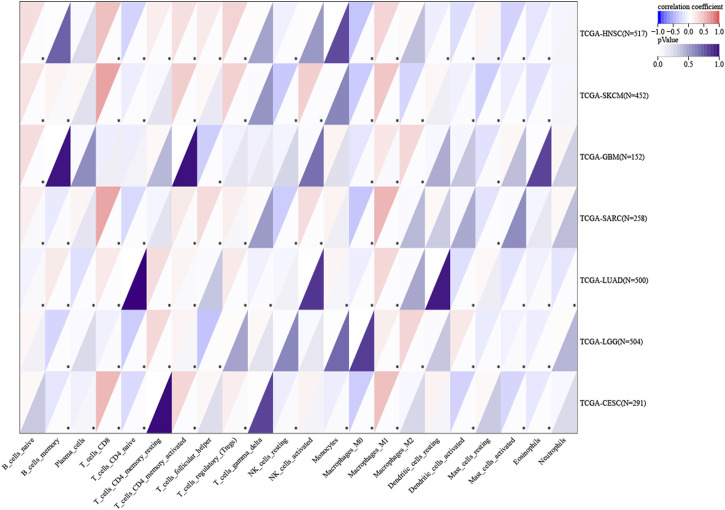
Association of ITGAL with the tumor microenvironment. Red highlights a positive correlation, and blue highlights a negative correlation; the darker the color, the stronger the association. (*p < 0.05, **p < 0.01, ***p < 0.001, ****p < 0.0001).

According to the data, five cancer types with high ITGAL expression predicted good prognosis (TCGA-CESC ^[N=273, p<0.05]^, TCGA-LUAD ^[N=490, p<0.05]^, TCGA-SARC ^[N=254, p<0.05]^, TCGA-HNSC ^[N=509, p<0.05]^, and TCGA-SKCM ^[N=444, p<0.05]^), while two cancer types with high ITGAL expression predicted poor prognosis (TCGA-LGG ^[N=504, p<0.05]^ and TCGA-GBM ^[N=152, p<0.05]^) (Figure 6). The immune cells such as CD8+T cells and activated CD4 memory cells had high infiltration in cancers where higher expression levels of ITGAL indicated good prognosis, while low CD8^+^ T cells’ infiltration was observed in two cancer types where high ITGAL expression was linked to poor prognosis. In all cancer types, NK cells exhibited low levels of infiltration. CD4 memory T cells showed high infiltration in LGG and LAML. M1 showed high invasion in all seven cancer types. Contralaterally, M2 showed high invasion in two cancer types with low ITGAL expression, indicating a good prognosis. Regulatory T cells (T-regs) invaded five cancer types with a p-value of 0.05 or higher. In seven cancer types among the species, the activated dendritic cells showed low invasion in most cancers but high invasion in LGG.

### ITGAL can be utilized as a biomarker for the detection of head and neck squamous cell carcinoma

**FIGURE 7:**
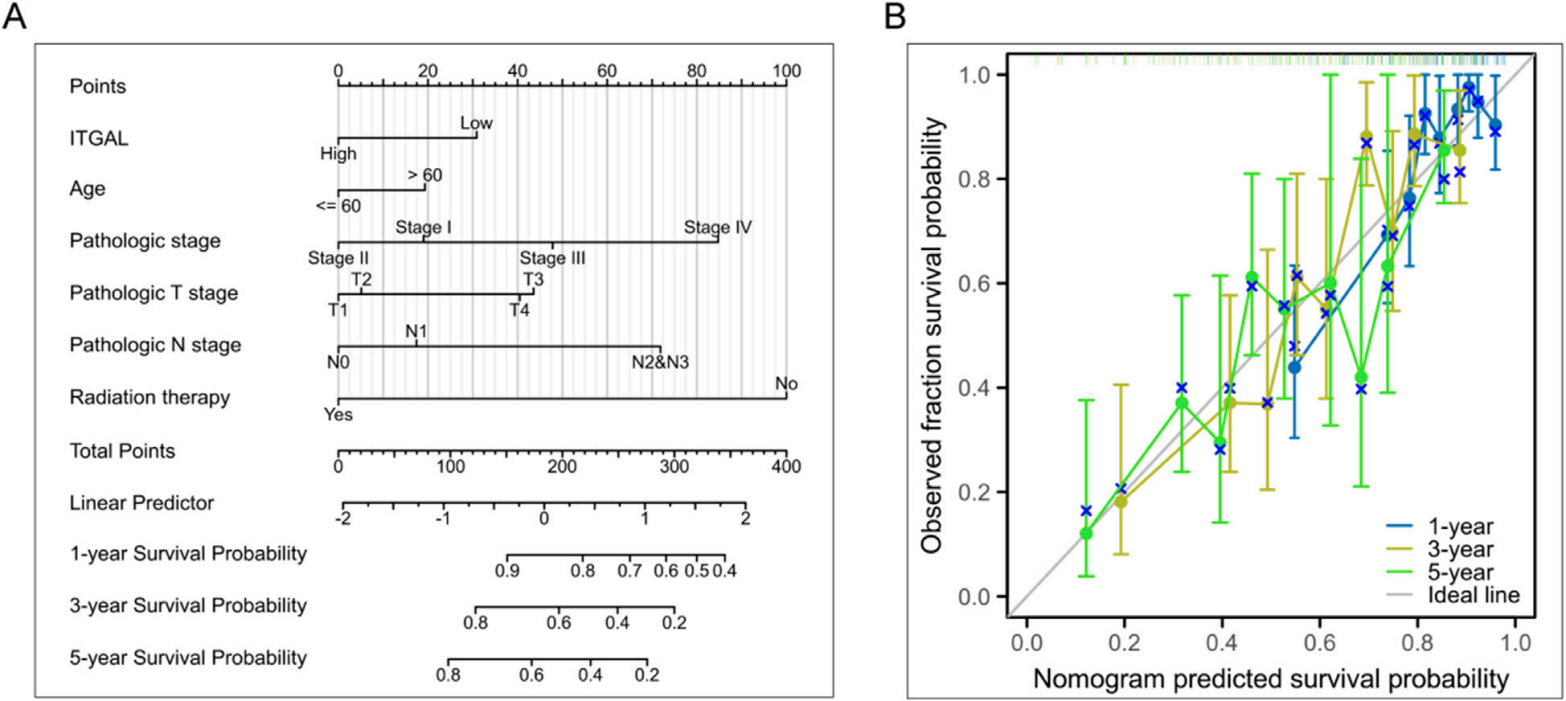
Investigation of the clinical significance of ITGAL in HNSCC. **(A)** Development of a nomogram utilizing ITGAL expression and pathological staging. **(B)** Prognostic standard curve of nomogram.

The studies revealed a substantial upregulation of ITGAL in HNSCC. Survival analysis further demonstrated a significant correlation between the expression level of ITGAL and the overall survival (OS) of HNSCC patients. Additionally, this correlation remained significant when examining the association between ITGAL and RNAss (RNA based Stemness Scores). Consequently, our subsequent investigation delved into the potential biological functions of ITGAL specifically within the context of HNSCC. In order to improve prognostic assessment for patients in clinical settings, we developed a nomogram that integrates the expression of ITGAL and the pathological stage. This nomogram provides a more accurate tool for predicting patient outcomes in HNSCC and can aid in clinical decision-making. The nomogram depicted in Figure 7A functions as a valuable instrument in clinical practice for foreseeing patient outcomes. Additionally, calibration curves were employed to evaluate the accuracy of the current model in forecasting the prognosis of patients with HNSCC at 1-year, 3-year, and 5-year intervals. The results, depicted in Figure 7B, demonstrate a favorable performance in the assessment of patient prognosis.

**FIGURE 8:**
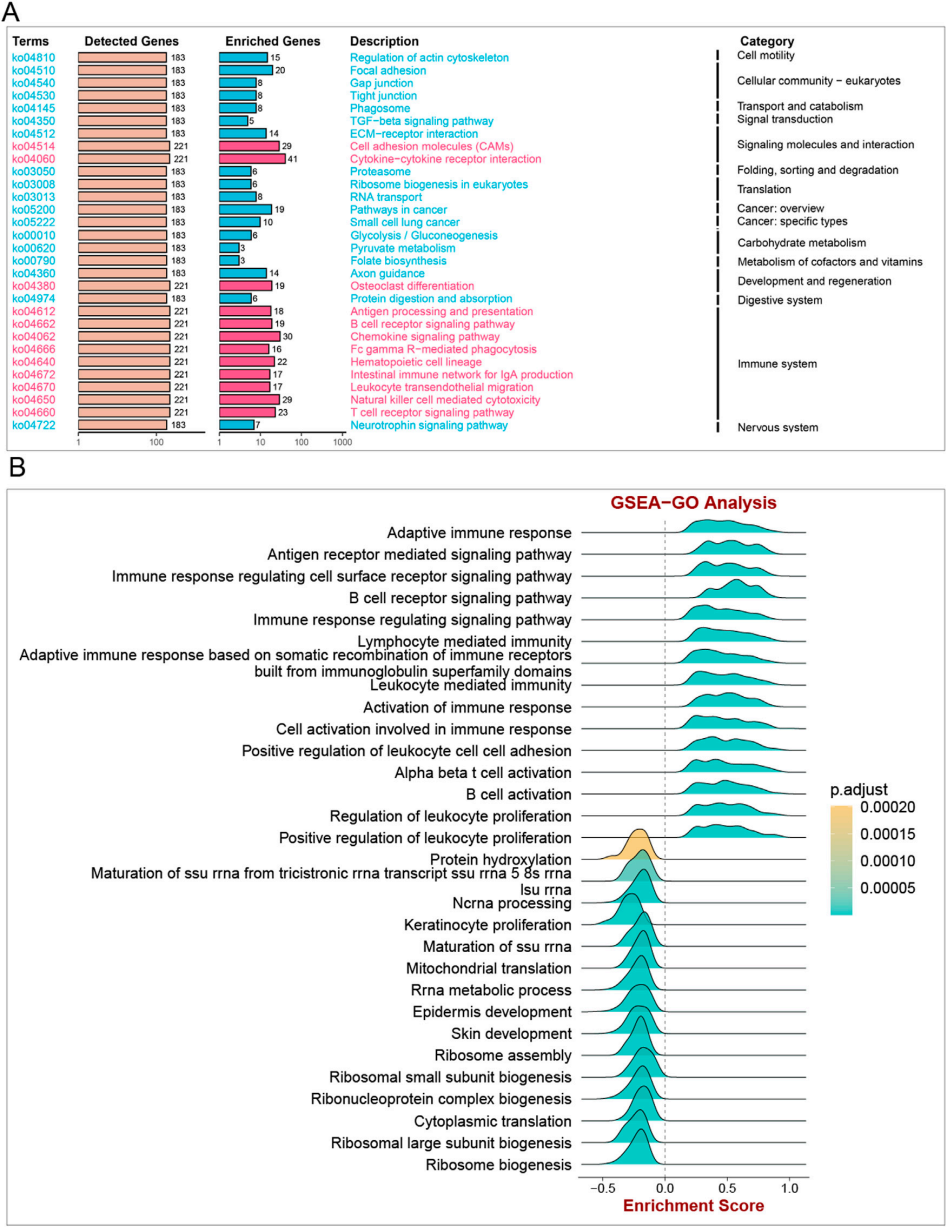
Enrichment analysis of ITGAL in HNSCC. **(A)** GSEA-KEGG analysis, **(B)** GSEA-GO analysis.

To gain deeper insights into the potential ways in which ITGAL affects patient prognosis, we conducted an analysis to determine the relationship between gene expression and pathway scores. Our findings indicate a significant correlation between ITGAL expression and various factors including cell motility, B cell activation, T cell activation, lymphocyte-mediated immunity, leukocyte-mediated immunity, leukocyte proliferation, cellular community, transport and catabolism, as well as carbohydrate metabolism in HNSCC (Figures 8A, B). Drawing from the GSEA results, we put forth the hypothesis that the influence on the malignant growth of HNSCC could potentially be accomplished by modulating signaling communication via the TGF-b pathway (Figure 8A).

### Link of the HNSCC signature with the TME

**FIGURE 9:**
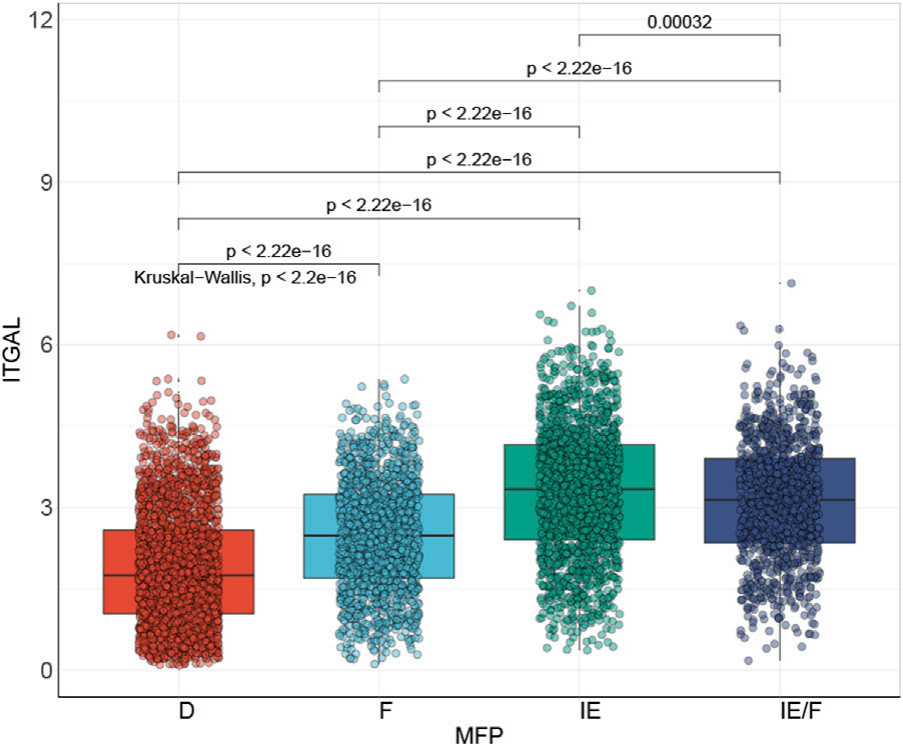
The association of ITGAL expression with tumor microenvironment subtype.

It has been suggested by several reports that TME is associated with the efficacy of immunotherapy (Ock et al., 2016; Varn et al., 2017; Bagaev et al., 2021). Bagaev et al. (2021), classified HNSCC immune microenvironment into four types: immune-depleted (D), fibrotic (F), immune-enriched (IE), and non-fibrotic and immune-enriched/fibrotic (IE/F) (Figure 9). His findings revealed that ITGAL expression was closely related to the, IE subtype, which is linked to a good prognosis.

### Single-cell analysis

**FIGURE 10:**
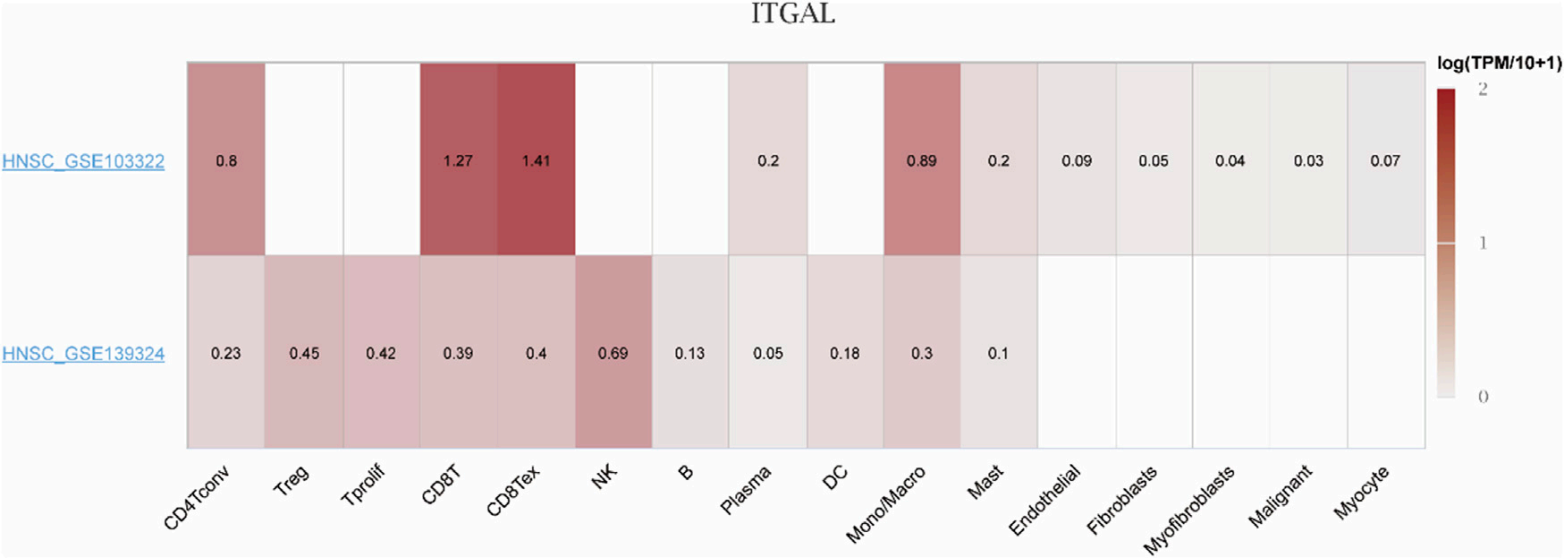
Correlation analysis between the expressions of ITGAL in HNSCC tissues and the TME, employing TISCH. Red indicates a positive correlation; the darker the color, the stronger the correlation.

**FIGURE 11:**
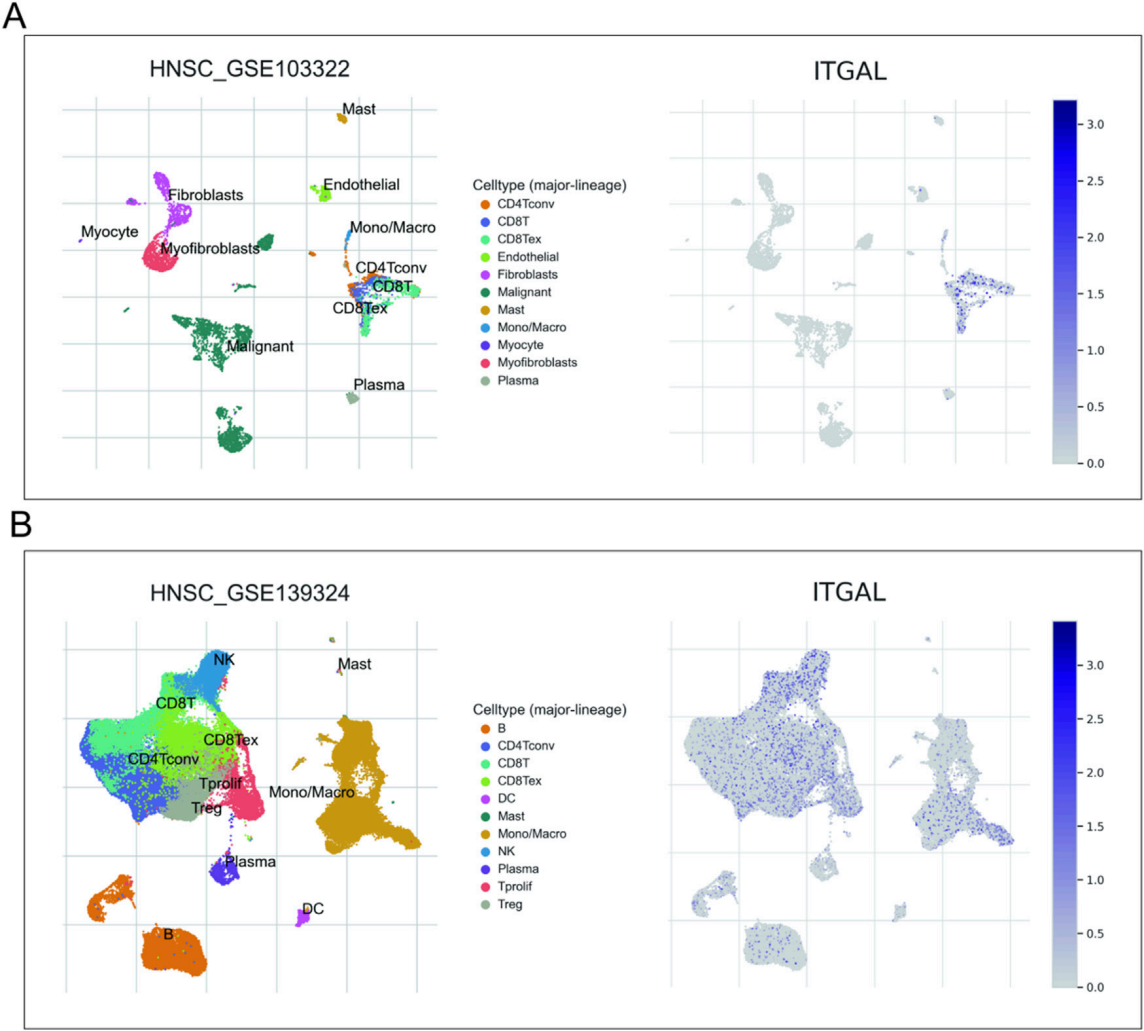
Correlation analysis between the expression of ITGAL in cancer tissues [GSE103322 **(A)** and GSE139324 **(B)**] and the TME utilizing TISCH.

Considering the contribution of TME to tumor onset and progression and its prognostic effect, HNSCC (GSE103322 and GSE139324) were analyzed in TISCH to assess the expression of ITGAL in TME-linked cells. The GSE103322 and GSE139324 datasets of HNSCC were analyzed and classified into 11 types of cells. CD8 T exhausted cells were the most abundant in the GSE103322 dataset. As indicated by Figure 10, the infiltration degree of ITGAL in TME-linked cells was higher in CD8+T, CD8 T exhausted, and CD4 conventional cells, which is in line with the findings presented in Figure 8B. In the GSE139324 dataset, the most abundant immune cells were CD4 conventional cells. The infiltration degree of ITGAL in TME-linked cells was higher in CD8+T, CD8 T exhausted, monocytes/macrophages, and B cells (Figure 11), which is in line with the findings in Figure 8B. It is suggested by these findings that ITGAL is closely linked to the TME in HNSCC.

### Pan-cancer analysis of ITGAL expression in correlation with the tumour purity, TMB, MSI, as well as stemness

**FIGURE 12:**
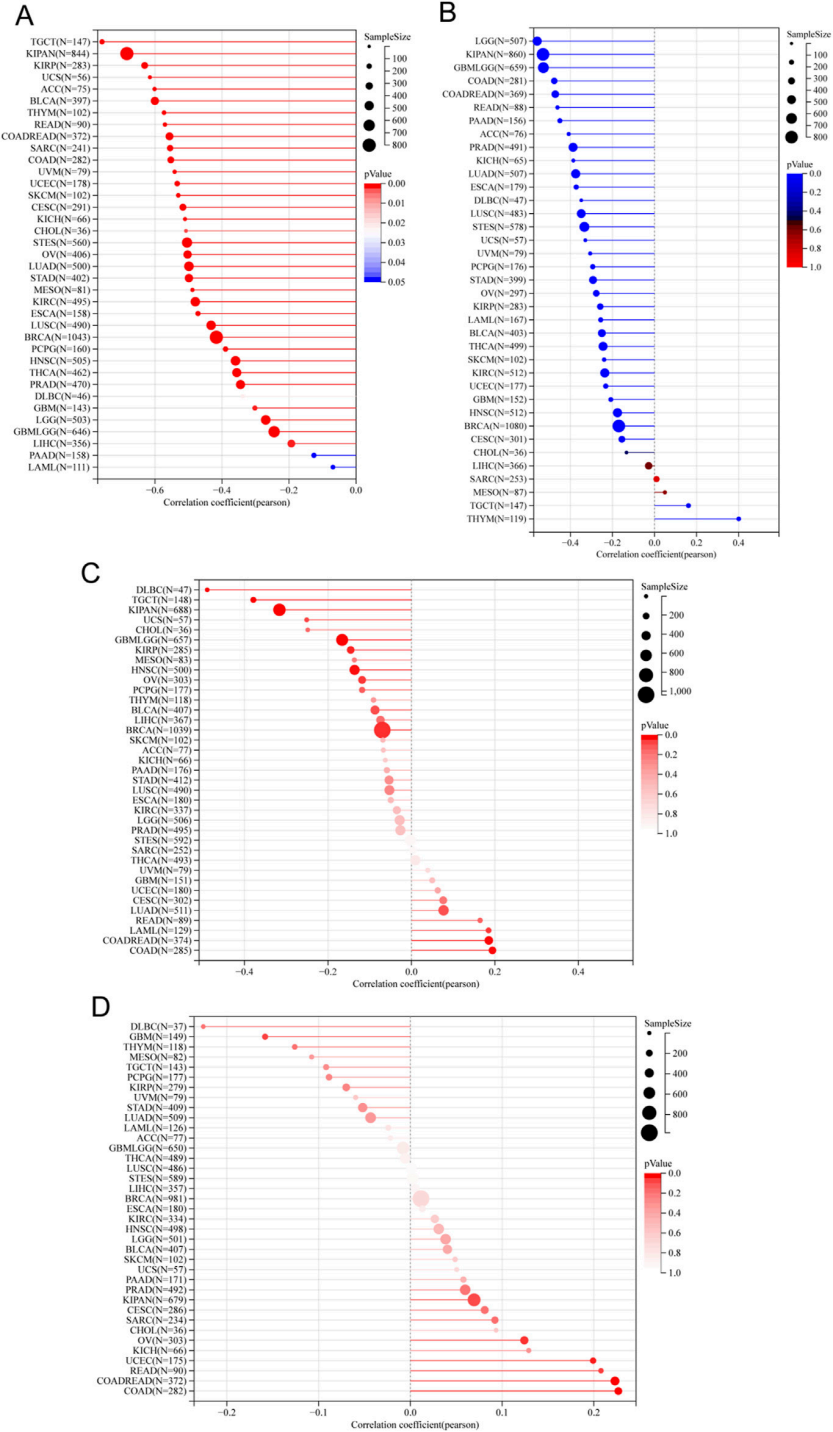
Correlation analysis of the association between ITGAL expression and tumour purity **(A)**, RNAss **(B)**, MSI **(C)** and TMB **(D)**.

In order to determine the suitability of immune checkpoint therapy, the correlation between ITGAL expression and TMB (Tumor Mutational Burden) as well as MSI (Microsatellite Instability) was investigated and compared across various cancer types. It was observed that TMB and MSI play a crucial role in this determination. Across various cancer types, the expression of ITGAL showed predominantly positive correlations with both TMB and MSI. Specifically, in COAD, COADREAD, UCEC, READ, OV, there was a significant positive association observed between the manifestation of ITGAL and TMB scores (Figure 12D). On the other hand, in patients with GBMLGG, BRCA, KIRP, KIPAN, HNSCC, OV, TGCT, DLBC, the expression of ITGAL displayed a closer and negative correlation with MSI. (Figure 12C). The effectiveness of immune checkpoint inhibitor (ICI) treatment can be influenced by tumor purity. During our analysis of 35 tumors, we identified a noteworthy negative correlation between the ITGAL expression and tumor purity. The observation made in our analysis indicates that there is a consistent association between higher levels of ITGAL expression and decreased tumor purity across all the tumor types that were examined (Figure 12A). Furthermore, the stemness score, which is associated with drug resistance and continuous tumor cell proliferation, was also evaluated in relation to ITGAL expression. In our study, we conducted a Pearson correlation analysis to investigate the relationship between ITGAL expression and RNAss across various tumors. The findings, depicted in Figure 12D, demonstrated significant correlations between ITGAL and tumor stemness scores in 32 tumor samples. Among these, 31 tumors exhibited a significant negative correlation. Notably, LGG (R = −0.55), KIPAN (R = −0.53), GBMLGG (R = −0.52), COAD (R = −0.47), READ (R = −0.46), PAAD (R = −0.45), and ACC (R = −0.40) were among the cancer types with the most significant correlations (Figure 12B). However, it is worth mentioning that THYM displayed a significant positive correlation in this relationship (R = 0.40).

### Immunotherapy response analysis

**FIGURE 13:**
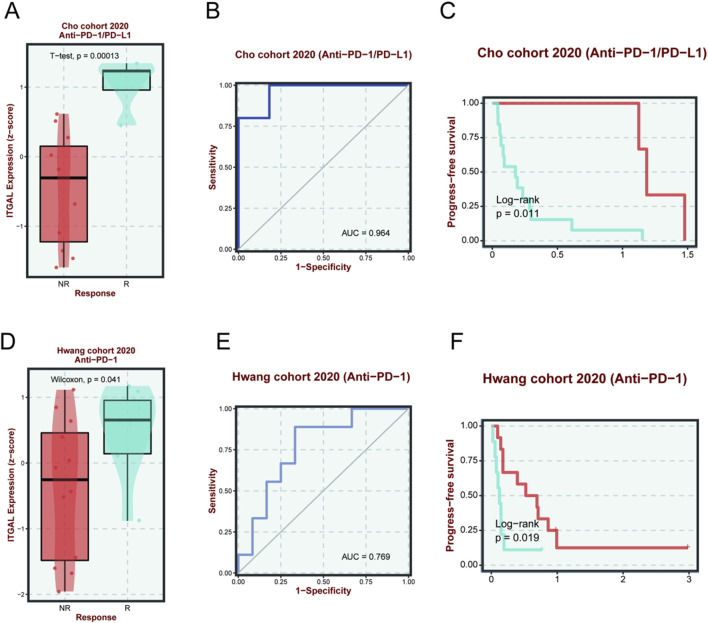
The relationship between ITGAL expression and immunotherapy sensitivity and prognosis of Cho cohort 2020 (Anti-PD-1/PD-L1) **(A–C)** and Hwang cohort 2020 (Anti-PD-1) **(D–F)**. The red line indicates a high expression of ITGAL, and the blue line indicates a lower expression of ITGAL.

We studied the drug treatment of HNSCC. In the Cho cohort 2020, it was discovered that the efficacy of Anti-PD-1/PD-L1 on HNSCC was closely related to the ITGAL expression. Those with high ITGAL expression were more efficient than the Anti-PD-1/PD-L1 (Figure 13A), and the probability was as high as 96.4% (Figure 13B). As depicted in Figure 13C, it could be seen that the PFS of those with high ITGAL expression was remarkably higher in comparison to the low expression group, confirming the role of ITGAL in the efficacy of Anti-PD-1/PD-L1. As per the cohort of Hwang 2020, we also found that the efficacy of Anti-PD-1 monotherapy on HNSCC was closely related to the ITGAL expression, and those with high ITGAL expression were more likely to respond to Anti-PD-1 (Figure 13D), and the probability was as high as 76.9% (Figure 13E). As shown in Figure 13F, the PFS of the ITGAL high-expression group was considerably higher in contrast with the low-expression group, confirming the role of ITGAL in the efficacy of Anti-PD-1. These findings revealed that the expression of ITGAL may be associated with the efficacy of Anti-PD-1/PD-L1 and Anti-PD-1 on HNSCC.

**FIGURE 14:**
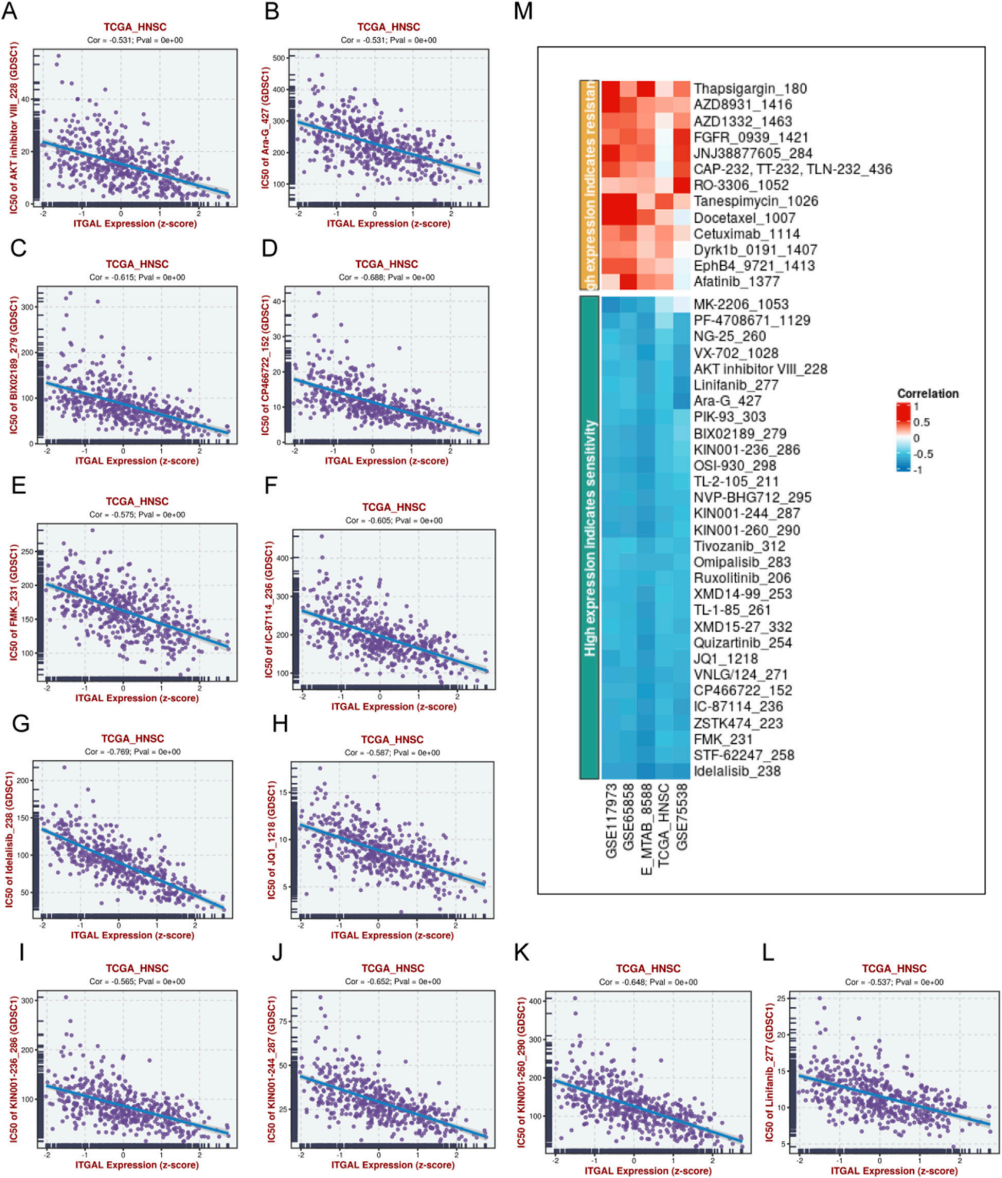
Potential drugs prediction of HNSCC. **(A)** IC50 of AKT inhibitor VIII_228, Ara-G_427 **(B)**, BIX02189279 **(C)**, CP466722_152 **(D)**, FMK_231 **(E)**, IC-87114_236 **(F)**, Idelalisib_238 **(G)**, JQ1_1218 **(H)**, KIN001-236_286 **(I)**, KIN001-244_287 **(J)**, KIN001-260_290 **(K)**, Linifanib_277 **(L)**,heatmap of the relationship between drugs sensitivity and ITGAL expression **(M)**.

In order to conduct a more thorough examination of potential drugs that could effectively target the overexpression of ITGAL, we conducted a comparison of the estimated IC50 levels for lots chemotherapy drugs or inhibitors in “GDSC1” database. Figure 14 displays a selection of representative drugs. We found that several drugs are potential for treating patients with ITGAL high. Expression, such as MK-2206_1053, PF-4708671_1129, NG-25 260, VX-702_1028, AKT inhibitor VIll_228, Linifanib_277, Ara-G 427, PIK-93303 and so on.In order to conduct a more thorough examination of potential drugs that could effectively target the overexpression of ITGAL, we conducted a comparison of the estimated IC50 levels for lots chemotherapy drugs or inhibitors in “GDSC1” database. Figure 13 displays a selection of representative drugs. We found that several drugs are potential for treating patients with ITGAL high Expression, such as MK-2206_1053, PF-4708671_1129, NG-25 260, VX-702_1028, AKT inhibitor VIll_228, Linifanib_277, Ara-G 427, PIK-93303 and so on.

## Discussion

Integrin alpha L chain encoded by ITGAL plays a crucial role in intercellular adhesion between leukocytes by binding to intercellular adhesion molecules 1–3 (ICAMs 1–3) (Corbi et al., 1988; Hickman et al., 2022). Furthermore, prior research suggests that LFA-1 encoded by ITGAL is closely associated with inflammatory responses, which are reduced significantly via the mechanism of blocking LFA-1 (Whitcup et al., 1999). These activities involve the interaction between leukocytes and endothelial cells, the killing of target cells by cytotoxic T-cells, and the killing of target cells through the assistance of antibodies by granulocytes and monocytes. It also promotes the cytotoxicity of natural killer cells (Barber et al., 2004).

We discovered that the expression of ITGAL in cancer tissues and paracancerous tissues is different in most cancer types, except UCEC, COAD, COADREAD, PCPG, CHOL. We conducted COX regression analysis and KM survival curves, which provided some confirmation that ITGAL has the potential to be a dependable biomarker. Upon comparing these results with those of the survival analysis, we discovered a significant association between ITGAL and four prognostic factors, namely OS, DSS, DFS, and PFS, across eight types of tumors, including GBMLGG, LGG, KIPAN, UVM, CESC, LUAD, HNSCC, SKCM. The potential of ITGAL as a biomarker is substantiated by its differential expression levels across distinct pathological stages within the same tumor. Increased expression of ITGAL is notably observed in the higher stages, further highlighting its significance as a potential biomarker. Immune cells and fibroblasts can exhibit both tumor-promoting and tumor-inhibiting effects within the microenvironment of a tumor. (Dudas, 2015), Improved understanding of the biological mechanisms that govern the tumor microenvironment (TME) could lead to more effective and targeted immunotherapies for various types of difficult-to-treat cancers, making it a valuable and potent tool in the fight against these diseases. At present, there are single-cancer immunoassay for ITGAL, but there is still a lack of an immunoassay for pan-cancer. Initially, we evaluated three immune scores, namely StromalScore, ImmuneScore, and EstimateScore, to determine their correlation with ITGAL in pan-cancer. Our findings indicated a strong positive correlation between ITGAL and these immune scores, indicating that ITGAL expression significantly contributes to the enhancement of immunity, which may be the reason why some cancers have a better prognosis, such as HNSCC, LUAD and SKCM. However, there are still many cancers with poor prognosis, including LGG, BGMLGG, KIPAN and UVM. Amanda’s study showed that ITGAL promotes Cx3cr1 expression, cx3cl1-mediated migration and Ccl5 expression in microglia, thereby promoting microglial infiltration and tumor formation (De Andrade Costa et al., 2021). Based on this study, we speculate that in KIPAN and UVM, ITGAL also increases the migration and invasion ability of cancer cells by affecting certain regulatory genes. The regulatory mechanism needs to be further studied and verified through experiments. In addition to that, we conducted a comprehensive analysis to determine the relationship between ITGAL and the infiltration of various immune cells. The results strongly suggest a notable association between the expression of ITGAL and multiple types of immune cells. Specifically, ITGAL expression positively influenced the infiltration of CD8^+^ T cells, Macrophages_M1 cells, and B cells, while inhibiting the infiltration of Macrophages_M2 cells. Prior research has demonstrated that the infiltration of CD8^+^ T cells can have a positive impact on the prognosis of patients (Liu et al., 2017), and high tumor stromal density of M2-like macrophages was associated with worse cancer-specific survival, which partly explains the improvement of prognosis of CESC, LUAD, HNSCC, SKCM with high expression of ITGAL. Moreover, the correlation linking ITGAL to immunomodulatory genes, including MHC, chemokines, and genes related to chemokine receptors, provides additional evidence of its association with tumor immunity. This association is bolstered by the observation that increasing ITGAL expression leads to a noticeable increase in MHC-1 expression. This is also one of the reasons why CESC, LUAD, HNSCC, SKCM have good prognosis when ITGAL is highly expressed.

The absence of immune cells within the tumor microenvironment has been linked to unfavorable outcomes in most cancer cases. This exclusion of immune cells is commonly observed alongside the presence of a stem cell-like characteristic, referred to as “stemness,” in tumors. The activation of a stemness program seems to hinder the body’s immune responses against the tumor through various mechanisms, such as the tumor cells themselves silencing endogenous retrovirus expression, suppressing type I interferon signaling, and increasing the expression of immunosuppressive checkpoints (Miranda et al., 2019). The study investigated the relationship between DNAss and ITGAL in different types of cancer, specifically LGG, UVM, LUAD, SKCM, and HNSCC. The findings revealed a positive correlation between ITGAL and DNAss in LGG and UVM, whereas a negative correlation was observed in LUAD, SKCM, and HNSCC. These results align with the prognostic outcomes, indicating that ITGAL potentially affects DNAss.

Head and neck squamous cell carcinoma ranks as the sixth most common cancer globally and holds the highest incidence in South Asia. This type of cancer accounts for approximately 890,000 newly reported cases and around 450,000 reported deaths worldwide (Kamal et al., 2023). We performed a focused analysis of the performance of ITGAL in HNSCC. And our analysis indeed revealed that ITGAL could serve as an independent prognostic factor for patients with HNSCC. And through online analysis, we looked for the possible action pathway of ITGAL, TGF-βsignaling pathway. The classification of HNSCC according to the effect of ITGAL on the immune microenvironment also shows that ITGAL can affect the prognosis of head and neck cancer through the immune microenvironment.

We used “IOBR” to analyze the infiltration degree of Immune cells in ITGAL. The results showed that high expression of ITGAL was positively correlated with the infiltration of CD8+T and Macrophages_M1 and the signature scores of CD8 T cells, T cell inflamed GEP, exhausted CD8, co-stimulation T, and inflamed T cells were remarkably higher. This increase indicated that ITGAL is closely related to T-cell immunity. Our findings suggested that ITGAL is critically involved in tumor immunity. To obtain a deeper understanding of the TME in HNSCC, we conducted a comprehensive analysis of cell types, annotating them at the single-cell level. We discovered that ITGAL was positively correlated with CD8+T, CD4+T, NK, and monocyte/macrophage infiltration in HNSCC, which can enhance immune killing against tumors.

Importantly, our research findings suggested that the efficacy of Anti-PD-1/PD-L1 and Anti-PD-1 on HNSCC was closely linked to the ITGAL expression; and those with high ITGAL expression were more likely efficient in Anti-PD-1/PD-L1. This finding may provide a targeted anti-tumor strategy for ITGAL to treat HNSCC. We further analyzed the effect of ITGAL on drug IC50, and found that with the increase of ITGAL expression, the IC50 of various drugs decreased, reflecting that ITGAL plays a positive role in the treatment of HNSCC.

Our findings may provide a targeted anti-tumor strategy for ITGAL by influencing the tumor immune microenvironment to treat HNSCC. Our study, however, has certain limitations. In this research, most of the data were retrieved from online databases, which are constantly updated and expanded; this may have influenced our research outcomes. Furthermore, we have not added information about complications. Third, whether immune cell infiltration correlates with the OS of patients was not determined in this research. This may provide an interesting research direction for further studies. While the present study has shed some light on the role of ITGAL in the realm of immunotherapy from a broad standpoint, it emphasizes the need for additional experimental investigations. The current findings provide strong indications for the importance of conducting further research in this area.

## Conclusion

In briefly, ITGAL acts as a pan-oncogene and displays distinct expression patterns in different types of cancer. These patterns provide valuable insights into patient prognosis and survival across various malignancies. It serves as a valuable therapeutic and prognostic indicator for diverse malignancies, particularly in HNSCC. Additionally, ITGAL displays noteworthy associations with infiltration of immune cell and immune checkpoint related genes, indicating its potential as a promising target in tumor immunotherapy. The findings from our research present a potential strategy to target ITGAL for anti-tumor purposes, with a specific focus on modulating the immune microenvironment of the tumor.

## Data Availability

The datasets presented in this study can be found in online repositories. The names of the repository/repositories and accession number(s) can be found in the article/Supplementary Material.

## Supplementary material

SUPPLEMENTARY FIGURE S1 GSEA of ITGAL in TCGA pan-cancer. The 15 leading significant pathways indicated by ITGAL GSEA results across the mentioned tumor types. Text in the same color represents the same signaling pathway in different cancers. **(A-F)**.

## Glossary

CESC: Cervical squamous cell carcinoma and endocervical adenocarcinoma
LUAD: Lung adenocarcinoma
SARC: Sarcoma
HNSCC: Head and Neck cell carcinoma
SKCM: Skin Cutaneous Melanoma
GBM: Glioblastoma multiforme
LGG: Brain Lower Grade Glioma
BLCA: Bladder Urothelial Carcinoma
CRC: Colon adenocarcinoma/Rectum adenocarcinoma Esophageal carcinoma
GBMLGG: Glioma
BRCA: Breast invasive carcinoma
ESCA: Esophageal carcinoma
STES: Stomach and Esophageal carcinoma
KIRP: Kidney renal papillary cell carcinoma
KIPAN: Pan-kidney cohort
STAD: Stomach adenocarcinoma
KIRC: Kidney renal clear cell carcinoma
LIHC: Liver hepatocellular carcinoma
OV: Ovarian serous cystadenocarcinoma
PAAD: Pancreatic adenocarcinoma
TGCT: Testicular Germ Cell Tumors
LAML: Acute Myeloid Leukemia
PRAD: Prostate adenocarcinoma
LUSC: Lung squamous cell carcinoma
WT: High-Risk Wilms Tumor
THCA: Thyroid carcinoma
READ: Rectum adenocarcinoma
UCS: Uterine Carcinosarcoma
ALL: Acute Lymphoblastic Leukemia
ACC: Adrenocortical carcinoma
KICH: Kidney Chromophobe
NSCLC: Non-small-cell lung cancer
CHOL: Cholangiocarcinoma
