# Supplementary figures and images for: Magnesium-related gene ITGAL: a key immunotherapy predictor and prognostic biomarker in pan-cancer
Source: Front Pharmacol. 2024 Nov 13;15:1464830. doi: 10.3389/fphar.2024.1464830 (PMC11598444; doi:10.3389/fphar.2024.1464830)

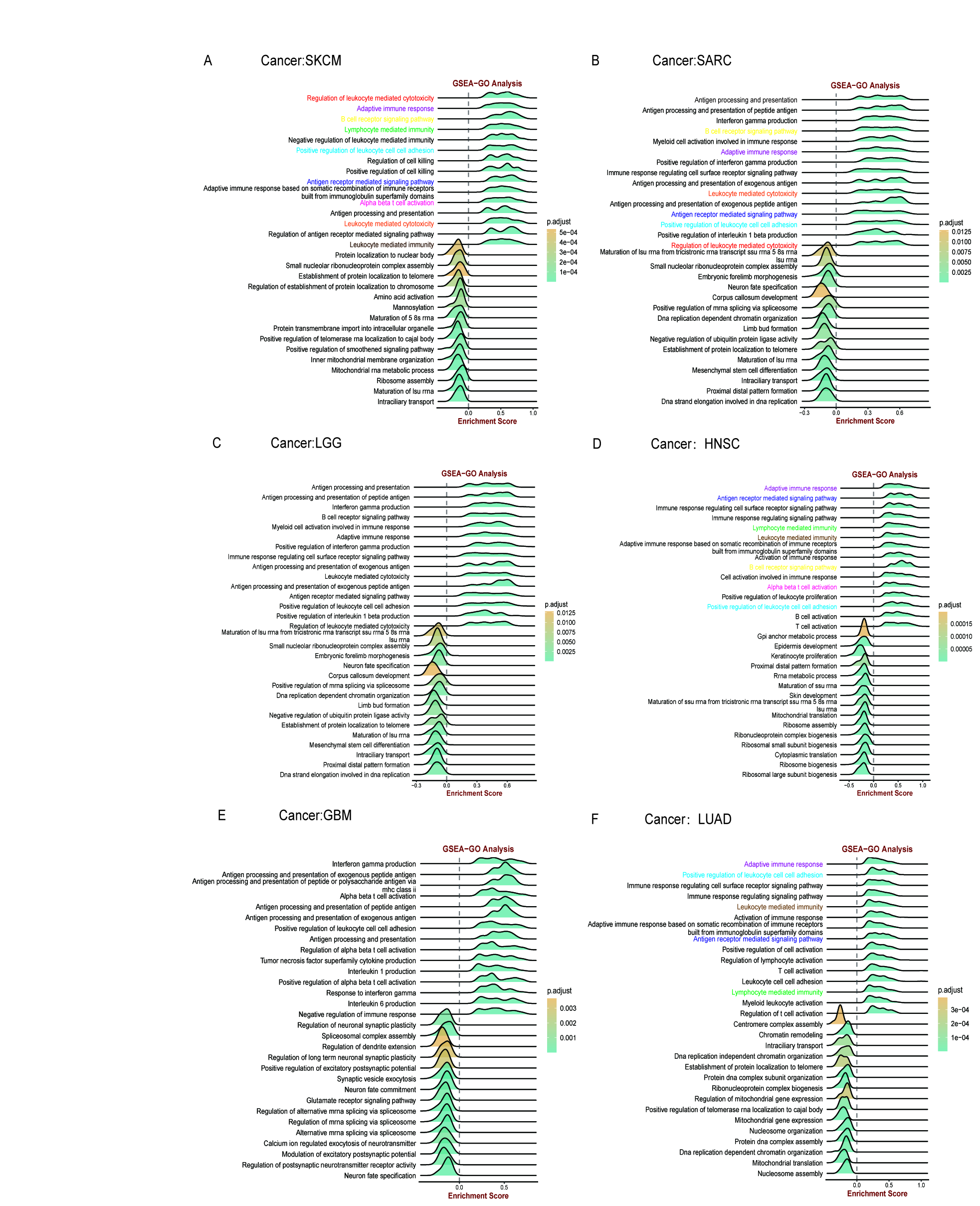

Supplement: Supplementary file 1 [file Image1.TIF]
